# Both Monoclonal and Polyclonal Immunoglobulin Contingents Mediate Complement Activation in Monoclonal Gammopathy Associated-C3 Glomerulopathy

**DOI:** 10.3389/fimmu.2018.02260

**Published:** 2018-10-02

**Authors:** Sophie Chauvet, Lubka T. Roumenina, Pierre Aucouturier, Maria-Chiara Marinozzi, Marie-Agnès Dragon-Durey, Alexandre Karras, Yahsou Delmas, Moglie Le Quintrec, Dominique Guerrot, Noémie Jourde-Chiche, David Ribes, Pierre Ronco, Frank Bridoux, Véronique Fremeaux-Bacchi

**Affiliations:** ^1^Assistance Publique-Hôpitaux de Paris, Hôpital Européen Georges Pompidou, Department of Nephrology, Paris, France; ^2^INSERM UMRS1138, Centre de Recherche des Cordeliers, Team “Complément et Maladies”, Paris, France; ^3^Université Paris Descartes Sorbonne Paris-Cité, Paris, France; ^4^Sorbonne Université, Paris, France; ^5^Assistance Publique-Hôpitaux de Paris, Hôpital Saint Antoine, Department of Immunology, Paris, France; ^6^INSERM UMRS 938, Sorbonne Universités, UPMC Univ Paris 06, Hôpital Saint-Antoine, Paris, France; ^7^Assistance Publique-Hôpitaux de Paris, Hôpital Européen Georges Pompidou, Department of Immunology, Paris, France; ^8^Department of Nephrology, Centre Hospitalier Universitaire de Bordeaux, Bordeaux, France; ^9^Department of Nephrology, Hôpital de Foch, Suresnes, France; ^10^Department of Nephrology, Centre Hospitalier Universitaire de Rouen, Rouen, France; ^11^Aix-Marseille Univ, UMRS 1076 Vascular Research Center of Marseille, Department of Nephrology, AP-HM, Marseille, France; ^12^Department of Nephrology, Centre Hospitalier Universitaire de Toulouse, Toulouse, France; ^13^Assistance Publique-Hôpitaux de Paris, Hôpital Tenon, Department of Nephrology, Paris, France; ^14^INSERM UMRS1155, Hôpital Tenon, Paris, France; ^15^Department of Nephrology, INSERM CIC 1402, Centre Hospitalier Universitaire de Poitiers, Poitiers, France; ^16^Centre National de Référence Maladies Rares: Amylose al et Autres Maladies à Dépôts d'Immunoglobulines Monoclonales, Université de Poitiers, Poitiers, France

**Keywords:** complement, alternative pathway activation, C3 glomerulopathies, monoclonal gammopathy, autoantibodies

## Abstract

C3 glomerulopathy (C3G) results from acquired or genetic abnormalities in the complement alternative pathway (AP). C3G with monoclonal immunoglobulin (MIg-C3G) was recently included in the spectrum of “monoclonal gammopathy of renal significance.” However, mechanisms of complement dysregulation in MIg-C3G are not described and the pathogenic effect of the monoclonal immunoglobulin is not understood. The purpose of this study was to investigate the mechanisms of complement dysregulation in a cohort of 41 patients with MIg-C3G. Low C3 level and elevated sC5b-9, both biomarkers of C3 and C5 convertase activation, were present in 44 and 78% of patients, respectively. Rare pathogenic variants were identified in 2/28 (7%) tested patients suggesting that the disease is acquired in a large majority of patients. Anti-complement auto-antibodies were found in 20/41 (49%) patients, including anti-FH (17%), anti-CR1 (27%), anti-FI (5%) auto-antibodies, and C3 Nephritic Factor (7%) and were polyclonal in 77% of patients. Using cofactor assay, the regulation of the AP was altered in presence of purified IgG from 3/9 and 4/7 patients with anti-FH or anti-CR1 antibodies respectively. By using fluid and solid phase AP activation, we showed that total purified IgG of 22/34 (65%) MIg-C3G patients were able to enhance C3 convertase activity. In five documented cases, we showed that the C3 convertase enhancement was mostly due to the monoclonal immunoglobulin, thus paving the way for a new mechanism of complement dysregulation in C3G. All together the results highlight the contribution of both polyclonal and monoclonal Ig in MIg-C3G. They provide direct insights to treatment approaches and opened up a potential way to a personalized therapeutic strategy based on chemotherapy adapted to the B cell clone or immunosuppressive therapy.

## Introduction

C3 glomerulopathy (C3G) is a heterogeneous group of rare glomerular diseases, characterized by predominant C3 deposition in glomeruli (1–3) and resulting from dysregulation of the complement alternative pathway (AP) (4–6). In physiological conditions, the complement AP is continuously activated at a low level and is amplified on activating surfaces, such as bacteria or dying cells (7). To avoid undesirable auto-amplification, the AP is tightly regulated in the fluid-phase and on cell surfaces by the plasma regulatory proteins factor H (FH), factor I (FI), membrane cofactor protein (MCP, CD46), complement receptor 1 (CR1, CD35), and decay accelerating factor (DAF, CD55) (8). Together, these regulators act by preventing the formation of and by dissociating the AP C3 convertase (FH, CR1, and DAF) and by serving as cofactors for FI-mediated inactivation of C3b to iC3b (FH, MCP, and CR1). Properdin is the only positive regulator of the AP, stabilizing the AP C3/C5 convertase (8, 9). Many of these factors are involved in complement dysregulation in C3G. Rare pathogenic variants in AP genes are identified in ~25% of C3G patients (5, 6, 10). In most cases, complement dysregulation is acquired, induced by the presence of Nephritic Factors (C3NeF and C5NeF), i.e., autoantibodies targeting the AP C3/C5 convertase (6, 10) or anti-FH antibodies (11, 12). Recently, C3G has been proposed to be included in the spectrum of monoclonal gammopathy of renal significance (MGRS) because of the high prevalence of monoclonal immunoglobulins (MIg) in C3G patients aged over 50, without criteria for multiple myeloma, that reached 30–71% in two small series, and 65% in the French C3G cohorts (13–16). Although this association and the favorable effect of clone-targeted therapy on renal outcomes (16) suggests a role of MIg in the occurrence of the renal disease, the exact pathophysiological link between MIg and AP dysregulation remains to be elucidated.

The aim of the current work was to determine the mechanism of acquired complement AP dysregulation in patients with MIg-C3G in order to clarify the causal relationship between the MIg and the occurrence of C3G.

## Methods

### Study population

Between 2000 and June 2014, 201 plasma samples from patients aged over 18 were received at the Laboratory of Immunology (European Hospital Georges Pompidou) for complement exploration in the context of C3G. The diagnosis of C3G was assessed by immunofluorescence according to consensus recommendations, with bright diffuse predominant C3 glomerular staining (≥2+), of at least two orders of magnitude greater than any other immune reactant (i.e., Ig). Patients with trace or weak amounts of IgM staining on glomerular sclerotic lesions were included, but those with weak staining for IgG, IgA or Ig light chains were excluded (2). The diagnosis of DDD was confirmed by demonstration of diffuse, highly electron-dense osmiophilic deposits within the lamina densa by EM. By contrast, the diagnosis of C3GN was established in patients showing deposits of lesser density without the characteristic distribution and “sausage shape” appearance of DDD (1). All patients with positive hepatitis B or C serology, antinuclear antigen autoantibodies, anti- double-stranded DNA antibodies, or cryoglobulinemia are excluded from the French C3G registry.

Search for monoclonal gammopathy was performed by immunofixation in all patients aged over 40. Of the 201 adult patients in the French registry of C3G, 60 patients (G1-G60) had a detectable MIg. Among them, 50 were included in a retrospective clinical study regarding the effect of chemotherapy on renal outcomes (16). In the current study, 41/60 MIg-C3G patients with available blood samples were included (Supplemental Figure 1). Two of 28 patients screened for genetic abnormalities carried a rare variant of undetermined significance (p.Asp130Asn in CFH and p.Glu548Gln in CFI), as previously described (16). As 96% of MIg-C3G patients displayed a C3GN pattern on kidney biopsy, 107 adult patients with C3GN without MIg extracted from the French cohort of C3G, and 8 patients with MIg without kidney disease, were used as control population. The local ethics committee approved the study and the study was approved by the Commission Nationale de L'informatique et des Libertés (CCP number 192 12 23) and all legal representative of children gave written informed consent for genetic analysis.

### Assays for complement component and for C3 and C5 nephritic factors

EDTA plasma samples were obtained from all patients. Plasma protein concentrations of C3, C4 were measured by nephelometry (Dade Behring, Deerfield, IL, USA). Soluble C5b-9 level determination was done using the MicroVue sC5b-9 Plus EIA Assay (Quidel, San Diego, CA), according to manufacturer instructions. Normal values were established from plasma samples from 100 healthy donors. C3NeF and C5NeF activities were determined by assessing the ability of purified plasma IgG to stabilize the membrane-bound C3bBb and C3bBbP convertases (6).

### ELISA detection for anti-FH, anti-FI, anti-CR1, anti-C3b and anti-FB antibodies

ELISA plates were coated with 10 to 15μg/ml of FH (11), FI, FB, C3b (17) (all from Complement Technologies, Tylor, Texas), CR1 (RandD System) in PBS for 1 h, followed by blocking of the plates with PBS-0.4% Tween 20. Plasma was diluted 1/200 in PBS-0.1% Tween 20 and applied for 1 h. Bound IgG or IgA was revealed by anti-human IgG antibody conjugated with HRP (Southern Biotech) or anti-human IgA antibody conjugated with HRP (Sigma) diluted in PBS-0.1% Tween 20, followed by TMB substrate system.

### Study of IgG binding to CR1 by surface plasmon resonance (SPR)

The interaction of patient IgG with CR1 was analyzed in real time using a ProteOn XPR36 SPR equipment (BioRad, Marne-la-coquette, France). CR1 (RandD System) was covalently immobilized to a GLC sensor chip (BioRad) following the manufacturer's procedure. Protein G purified IgG from the patients or healthy donors (at 100 μg/ml) were injected for 300 s in PBS 0.005% Tween 20 containing running buffer. The dissociation was followed for 300 s. The signal from the interspots, reflecting the background binding was subtracted, as recommended by the manufacturer.

### Study of C3b interaction with CR1 in presence of patients IgG by surface plasmon resonance

IgG from patients with anti-CR1 antibodies were tested for their capacity to alter the C3b binding to CR1 using SPR. CR1 was coupled to individual flow channels of GLC biosensor chip using standard amine-coupling, according to the manufacturer's instruction. Total purified IgG were flowed at a concentration 100 mg/ml followed by injection of C3b (Complement Technologies) at concentrations starting from 1 μg/ml. Five concentrations and a running buffer were injected at 30 μl/min in HEPES buffer (10 mM Hepes, 25 mM NaCl, Tween 0.005%, pH 7.4) for 300 s across the immobilized ligand. Data were analyzed using ProteOn Manager software and the data from the blank channel were subtracted. Kinetic parameters were calculated by fitting the obtained sensorgrams into a two-state interaction model.

### Determination of light chain and heavy chain isotype specificity of anti-complement protein antibodies

The light chain (LC) isotype of antibodies was determined by ELISA. After plasma incubation and washing, isotype-specific goat antibodies directed against kappa and lambda LC (Southern Biotech), diluted in PBS-0.1% Tween 20 were incubated 1 h. Bound Ig was revealed by a Rabbit anti goat IgG Ab (Santa Cruz) diluted in PBS-0.1% Tween 20, followed by TMB substrate system. The ratios of the optical densities obtained with the anti-k and anti-l Abs (k/l) were calculated for all samples. A k/l ratio <0.1 or >3 indicated the predominance of anti-complement autoantibody of lambda or kappa LC specificity respectively, as previously described. A ratio between 0.1 and 3 indicated both kappa and lambda reactivity (11). The heavy chain (HC) IgG subtypes of anti FH and anti CR1 IgG Ab were determined by an anti-FH or anti-CR1 ELISA. After plasma incubation and washing, isotype-specific mouse antibodies directed against IgG1, IgG2, IgG3, and IgG4 (NL16 for IgG1, GOM2 for IgG2, ZG4 for IgG3, and RJ4 or IgG4) (Unipath, Bedford, UK), diluted PBS-0.1% Tween 20 were incubated 1 h. Bound IgG was revealed by a rabbit anti mouse IgG Ab (Jackson ImmunoResearch) diluted in PBS-0.1% Tween 20, followed by TMB substrate system.

### Determination of LC and HC isotype specificity of monoclonal immunoglobulin

The analysis of serum MIg of 29/41 patients was performed by a western blotting. Serum dilutions were adjusted to normalized gamma globulin levels. Proteins were separated by high-resolution thin layer agarose electrophoresis and transferred on nitrocellulose sheets. After saturation with skimmed milk, the blots were probed with polyclonal antibodies specific for a, g, m, k or l Ig chains or with monoclonal antibodies specific for IgG subclasses with NL16 for IgG1, GOM2 for IgG2, ZG4 for IgG3, and RJ4 or IgG4 (Unipath, Bedford, UK), followed by peroxydase coupled rabbit anti mouse IgG antibodies (Jackson ImmunoResearch). The signal was developed by chemo luminescence using ECL kit (Perkin Elmer) and MyECL Imager (Thermo Scientific).

### IgG purification

#### Total IgG purification

IgG were purified from plasma of MIg-C3G patients or from plasma of control patients (healthy donors, patients with positive C3NeF and patients with MIg but without kidney disease) by using Protein G beads (GE Healthcare), as recommended by the manufacturer. The concentration of the IgG was determined by a Nanodrop spectrophotometer.

#### Purification of monoclonal and polyclonal Igs by chromatography

Monoclonal and polyclonal Ig fractions of 5 patients (with monoclonal IgG) were purified using ion exchange column chromatography. Each plasma sample was dialyzed against 10 mM Tris (pH8). Prepaked diethyl-aminoethyl (DEAE) trisacryl column (Life Science) was equilibrated with 10 mM Tris (pH8). The dialyzed samples were loaded onto the column followed by elution with a 0–0.2 M NaCl gradient in 10 mM Tris buffer (pH8). Serial 1 ml fractions were collected and assayed for protein concentration (280 nm OD). The fractions were tested by agarose electrophoresis and immunofixation to determine which fractions contained polyclonal or MIg.

### Cofactor assays

C3 protein (20 μg/ml; Calbiochem) was incubated at 37°C for 0, 1, 5, or 10 min with FI (10 μg/ml; Complement Technologies) and FH (20 μg/ml; Complement Technologies, Tylor, Texas), or soluble CR1 (10 μg/ml; RandD Systems) in 10 mM Tris, 150 mM NaCl, pH 7.4 in presence of 100 μg/ml of total purified IgG. Samples were boiled and the cleavage of the C3 was probed by a Western blot, using SNAP system (Millipore). After blocking with Tris 10 mM, NaCl 150 mM, 0.1% Tween, 1% BSA, the blots were probed with a 1:5,000 dilution of goat anti-human C3 IgG (Calbiochem) followed by HRP-conjugated rabbit anti-goat IgG (Santa Cruz). The signal was developed by chemiluminescence using ECL kit (Perkin Elmer) and MyECL Imager (Thermo Scientific). Cleavage efficiency was evaluated by the appearance of the α43 band and the disappearance of the α-chain at 10 min and quantitated by densitometry of the scanned images. The ratio between α43 and the β bands (representing the % of C3b cleaved) was plotted vs. the time of incubation.

### C3 convertase formation in normal human serum in presence of patients' IgG

Purified total IgG from patients or healthy donors were incubated for 30 min at 37°C with normal human serum diluted 1:3 in presence of EGTA-Mg to block the classical pathway (10 mM MgCl_2_,10 mM EGTA, 40 mM NaCl Hepes buffer). The generation of C3a was quantified by the Micro Vue C3a Kit (Quidel) according to the manufacturer's instructions. IgG from 8 patients with MIg without kidney disease were used as controls.

### Fluid phase C3 convertase activation in presence of patients' IgG

Total purified IgG (100 μg/ml) were incubated for 45 min at 37°C with C3 (25 μg/ml), FB (0 to 50 ng), FD (0.05 μg/ml) (all from Complement Technologies, Tylor, Texas) in Hepes, 40 mM NaCl supplemented with 10 mM MgCl_2_. The reaction was stopped by adding DTT-containing sample buffer. The cleavage of C3 was probed by a Western blot, using SNAP system (Millipore). After blocking with Tris 10 mM, NaCl 150 mM, 0.1% Tween, 1% BSA, blots were probed with a 1:5,000 dilution of goat anti-human C3 IgG (Calbiochem) followed by HRP-conjugated rabbit anti-goat IgG (Santa Cruz). The signal was developed by chemiluminescence using ECL kit (Perkin Elmer) and MyECL Imager (Thermo Scientific). Percentage of C3 cleavage revealing convertase formation was characterized by the appearance of α'-band and quantitated by densitometry of the scanned images. The ratio between α' and the β bands was calculated at 50 ng of FB. The same experiment was reproduced with monoclonal and polyclonal Ig fractions of 5 patients and with IgG of 3 MIg-C3GN patients after chemotherapy adapted to the B cell clone. IgG from 8 patients with MIg without kidney disease were used as controls. The same experiment was reproduced with monoclonal and polyclonal fractions of 5 MIg-C3G patients IgG.

### C3 convertase activation on immobilized patient IgG

Coating of ELISA plate was performed at 20μg/ml of purified IgG in PBS for 1 h followed by a blocking of the plates by PBS-0.4% Tween 20. After washing, C3 convertase was formed by adding C3 (25 μg/ml), FB (0–50 ng), FD (0.05 μg/ml) (all from Complement Technologies, Tylor, Texas) diluted in Hepes, 40 mM NaCl supplemented with 10 mM MgCl_2_. The cleavage of C3 was probed by a Western blot and quantified as described above.

### Statistical analyses

Data are expressed as median (with range) for continuous variables and percentage for categorical variables. Statistical analyses were performed using the Mann-Whitney and Kruskal-Wallis tests, as appropriate, for comparison of continuous variables. Chi-square or Fisher's exact tests were used for comparison of categorical variables. *P*-values below 0.05 were considered significant. Results were analyzed using the Graph Pad Prism software.

## Results

### MIg-C3G is associated with biomarkers of C3/C5 convertase activation

**Figure 1:**
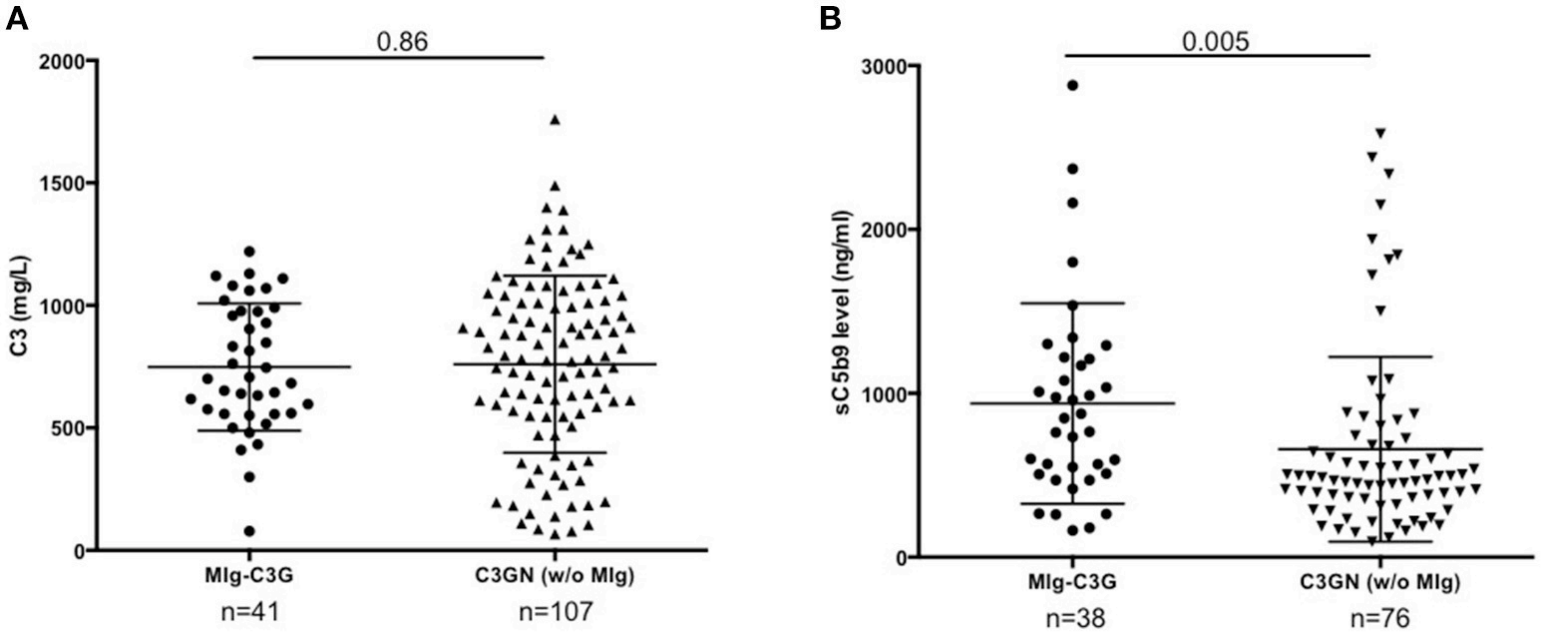
C3 and s C5b9 levels in C3G patients. **(A)** Plasma levels of C3 and **(B)** sC5b-9 of 41 patients with MIg-C3G (including 39 with C3GN and 2 with DDD pattern) and adult C3GN patients without MIg (*n* = 107). For measurement of sC5b9, 76/107 C3GN patients were tested. Fifty healthy controls were used to calculate the normal range of sC5b-9 (below 460 ng/ml). The mean ± standard deviation of each group is indicated.

Forty-one patients from the French registry of C3G met inclusion criteria (Supplemental Figure 1). Baseline clinical data and complement biomarkers are detailed in Supplemental Table 1 and Table 1. At diagnosis, 18/41 (44%) MIg-C3G patients had a low C3 level and a normal C4 (Table 1). Median C3 level of MIg-C3G patients and C3GN patients without MIg were similar (*p* = 0.86) (Figure 1A). Soluble C5b-9 was increased in 29/37 (78%) MIg-C3G patients and in 47/76 (62%) C3GN patients without MIg (*p* = 0.09) (Table 1). Median sC5b-9 level was significantly higher in MIg-C3G patients compared to patients without MIg (*p* = 0.005) (Figure 1B).

**Table 1:** Comparison of immunological findings in 41 MIg-C3G patients and 107 C3GN adults patients without MIg.

	**MIg-C3G**	**Adults C3GN**	***p-*value**
	***N* = 41**	***N* = 107**	
**IMMUNOLOGICAL FINDINGS**			
C3 (mg/L)	703 (78-1220)	781 (67-1760)	0.86
Low C3 level, n(%)	18 (44%)	56 (40%)	0.71
C4 (mg/L)	250 (104-575)^*^	252 (94-751)^*^	1
sC5b-9 (ng/mL)	848 (164-2880)	478 (94-2582)	0.005
Elevated sC5b9 (upper 420ng/mL)	29/37 (78%)	47/76 (62%)	0.09
Elevated sC5b-9 (upper twice the normal)	15/37 (41%)	13/76 (17%)	0.01
C3NeF, n(%)	3 (7%)	44/98 (45%)	0.0001
C5NeF, n(%)	0/12 (0%)	11/21(52%)	0.002
Anti-FH Abs, n(%)	9 (17%)	10/91 (11%)	0.09
Anti-FI Abs, n(%)	2 (5%)	NA	-
Anti-CR1 Abs, n(%)	11 (27%)	3/84 (4%)	0.0001
**GENETIC ANALYSIS**			
Pathogenic variants	2/28(7%)	27/99 (27%)^**^	0.02

* C4 level was normal in all patients

** *99 on 107 C3GN patients without monoclonal gammopathy were screened for genetics abnormalities of complement proteins. Results are described in Servais et al. (4) and Marinozzi et al. (6)*.

### Detection of anti-complement protein auto-antibodies in MIg-C3G

**Figure 2:**
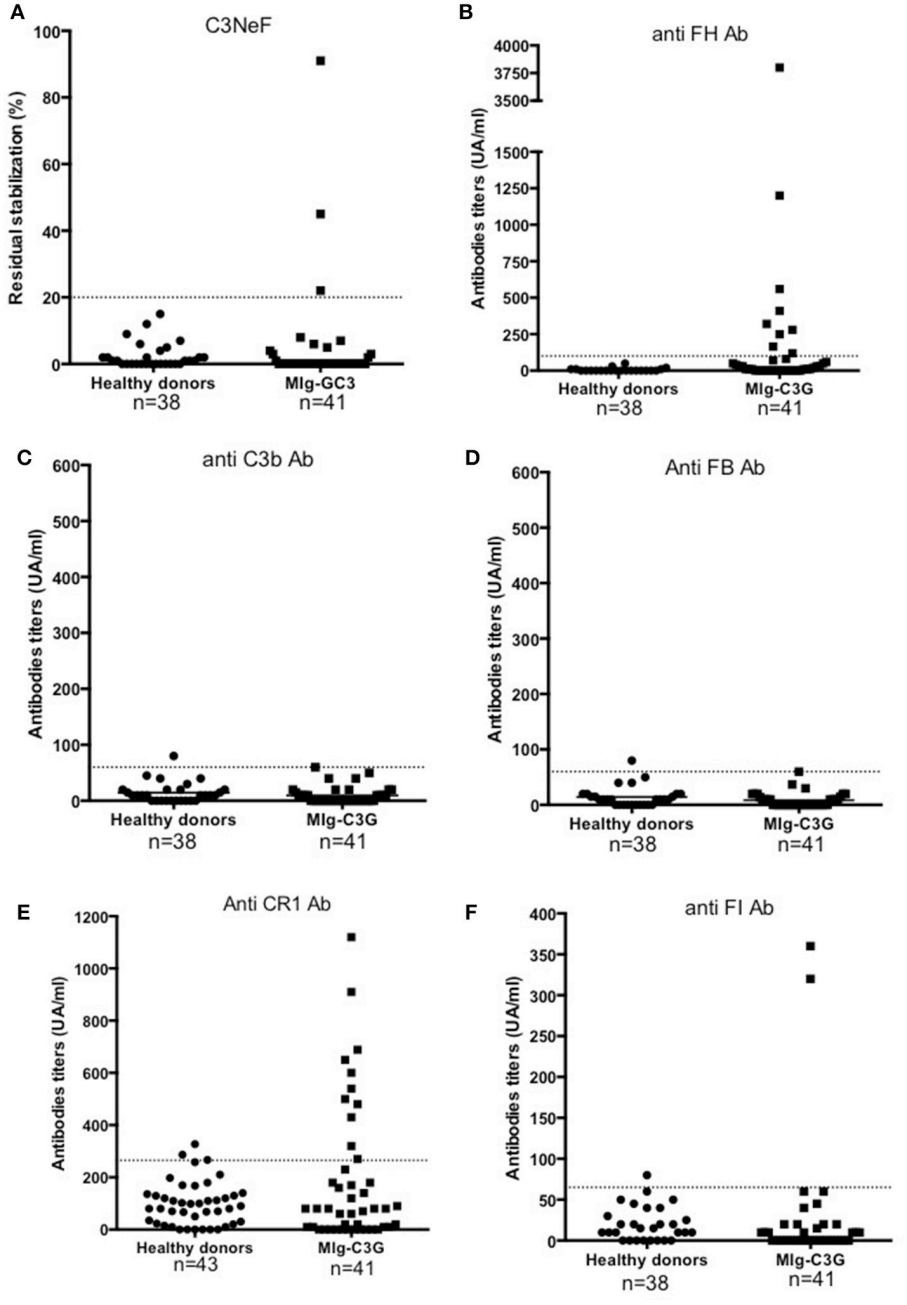
Detection of auto-antibodies against complement proteins. **(A–F)** Reactivity of Ig in plasma samples against FH, C3b, FB, CR1, FI, and C3 convertase (C3NeF assay). Samples from 41 MIg-C3G patients and 38 healthy individuals were tested. Results of C3NeF and other antibodies are expressed as percentage of residual stabilization, and in arbitrary units (UA), respectively. For anti-FH, anti-C3b and anti-FB antibodies, we used positive controls as previously described (one patient positive for anti-FH auto-antibodies in the setting of atypical HUS and one patient positive for both anti-C3b and anti-FB auto-antibodies) (11, 17). For the other ELISA assays, results were considered as positive when the OD was upper the mean +2SD (of the OD obtained with IgG from healthy donors). The patient's sample with the higher OD value was then used to determine the UA.

Samples were screened for C3 NeF/C5 NeF and auto-antibodies targeting 5 proteins of the AP (Figures 2A-F). Anti-FH auto-antibodies, C3NeF and anti-FI auto-antibodies were detected in 17% (9/41), 7% (3/41), and 5% (2/41) of MIg-C3G patients, respectively. None had anti-C3b, anti-FB antibodies or C5NeF. Eleven patients were positive for anti-CR1 auto-antibodies (11/41, 27%) (Figure 2E and Supplemental Figure 2A). The characteristics of the binding of anti-CR1 positive IgG to CR1 by Surface Plasmon Resonance (SPR) are provided (Supplemental Figures 2B-C). Overall, anti-complement auto-antibodies were detected in 20/41 (49%) MIg-C3G patients, including 4 patients with combined anti-FH and anti-CR1 antibodies and 1 with anti-FI and anti-FH antibodies.

**Figure 3:**
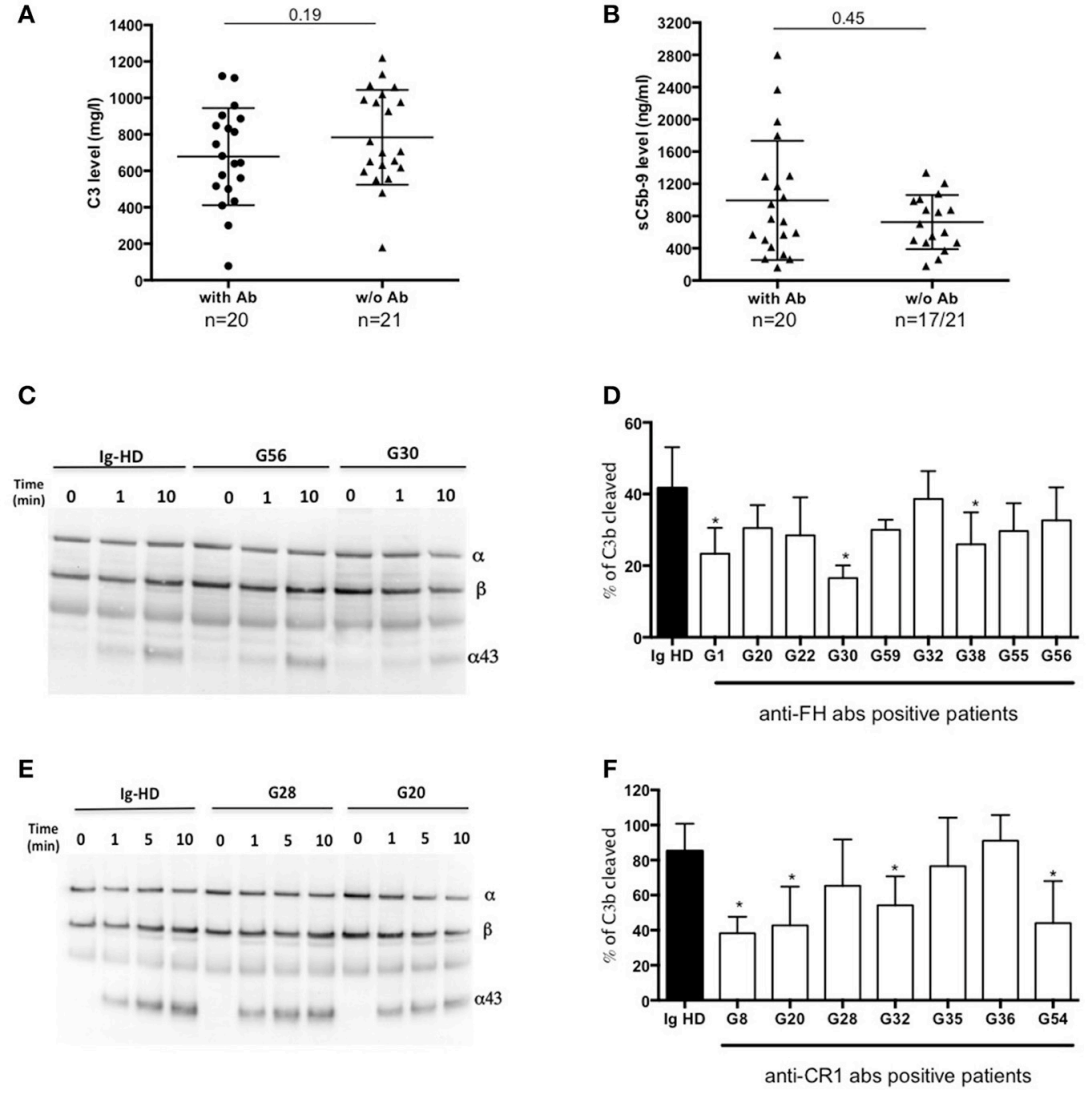
Functional consequences of anti-complement antibodies. **(A)** Plasma levels of C3 and **(B)** sC5b-9 of 22 MIg-C3G patients with anti-complement protein antibodies and 19 MIg-C3G without antibodies. **(C-D)** Analysis of Factor I-dependant cofactor activity of FH in presence of IgG from patients positive for anti-FH antibodies and **(E-F)** of CR1 in presence of IgG from patients positive for anti-CR1 antibodies. Cleavage of C3b to iC3b was indicated by the generation of α43 fragment and decrease of the α-chain. The percentage of C3b cleaved was determined by the ratio α43/β chain (*n* = 3 experiments). ^*^p < 0.05.

C3 and sC5b9 levels were similar in patients with or without antibodies (Figures 3A,B). Compared to C3GN patients without MIg, MIg-C3G patients had significantly lower frequency of C3NeF [3/41(7%) vs. 44/98(45%); *p* = 0.0001] and C5NeF (*p* = 0.002), higher frequency of anti-CR1 auto-antibodies [11/41(27%) vs. 3/84(4%); *p* = 0.0001] and similar frequency of anti-FH auto-antibodies (Table 1).

Functional studies were carried out in patients with anti-FH, anti-CR1 and anti-FI antibodies. We studied the impact of anti-FH antibodies on AP regulation by studying the capacity of FI to cleave C3b in iC3b in presence of FH. We performed a fluid phase cofactor assay in presence of total IgG purified from patients with anti-FH antibodies or healthy donors (HD). The C3b cleavage was revealed by Western Blot and ratio α43-chain on β-chain of C3b, determined by densitometry, was used to determine the % of C3b cleavage. C3b cleavage was significantly decreased in 3/9 patients with anti-FH antibodies (Figures 3C,D).

We next studied the functional properties of anti-CR1 antibodies. The presence of anti-CR1 antibodies resulted in decreased capacity (from 12 to 25%) of C3b to bind CR1, as demonstrated by SPR-based technology (Supplemental Figures 2D–F). Moreover, by Western blot, significant reduced CR1 cofactor activity for FI was obtained in presence of IgG purified from 4/7 anti-CR1 positive patients (Figures 3E,F). In 2 patients with anti-FI antibodies, C3b cleavage by FI in presence of FH was not decreased (data not shown).

### Study of light and heavy chain isotype specificity of anti-complement protein antibodies

MIg heavy chain (HC) and light chain (LC) isotype specificities were determined by immunoblot in 29 patients (Supplemental Table 2).

Using ELISA, we determined heavy chain (HC) and light chain (LC) isotype specificity of anti-complement antibodies in 13 positive patients. In 3 cases, anti-FI IgA, anti-FH IgG or anti-FH IgA antibodies displayed similar HC and LC restriction as the MIg. In 10/13 (77%) positive patients, the MIg HC (all of IgG isotype) and/or LC did not match those of the respective auto-antibodies (Table 2).

**Table 2:** Heavy and light chain characterization of anti-complement protein Ab and monoclonal immunoglobulin.

**Patient**	**MIg**	**Anti-complement protein Ab**			**Similar HC and LC specificity between Ab and MIg**
		**Spécificity**	**HC**	**LC**	
G30	IgG1λ	Anti FH	γ3	κ and λ	No
G22	IgG4k	Anti FH	γ2	κ and λ	No
G38	IgG2k	Anti FH	γ2	κ	Yes
G55	IgAk	Anti FH	α	κ	Yes
G8	IgG4l	Anti CR1	γ1	κ and λ	No
G13	IgG1k	Anti CR1	γ1	κ and λ	No
G15	IgG1λ	Anti CR1	γ1, γ4	κ and λ	No
G35	IgG3k	Anti CR1	γ1	κ and λ	No
G28	IgG2k	Anti CR1	γ1	κ and λ	No
G54	LCk	Anti CR1	γ1	κ and λ	No
G40	IgAk	Anti FI	α	κ	Yes
G20	IgG2λ	Anti FH/CR1	Anti-FH γ1 and	κ and λ	No
			Anti-CR1		
G32	IgG4l	anti FH/anti CR1	Anti-FH γ2 and	κ and λ	No
			Anti-CR1 γ1		

Abbreviations: Ab, antibody, HC: heavy chain, LC: light chain, MIg: monoclonal immunoglobulin

### Patients' Ig induce fluid-phase and solid-phase AP convertase activation

To test the capacity of total purified IgG (containing the MIg) of MIg-C3G patients to activate complement AP, we measured C3a release in normal human serum (NHS) by ELISA after incubation with patients' IgG or IgG from healthy donors (Supplemental Figure 3). For 10/32 patients' IgG, C3a level was above the mean+2SD cut-off obtained with IgG from healthy donors (Supplemental Figure 3A). C3a release was similar in MIg-C3G patients with or without anti-complement protein auto-antibodies (Supplemental Figure 3B).

**Figure 4:**
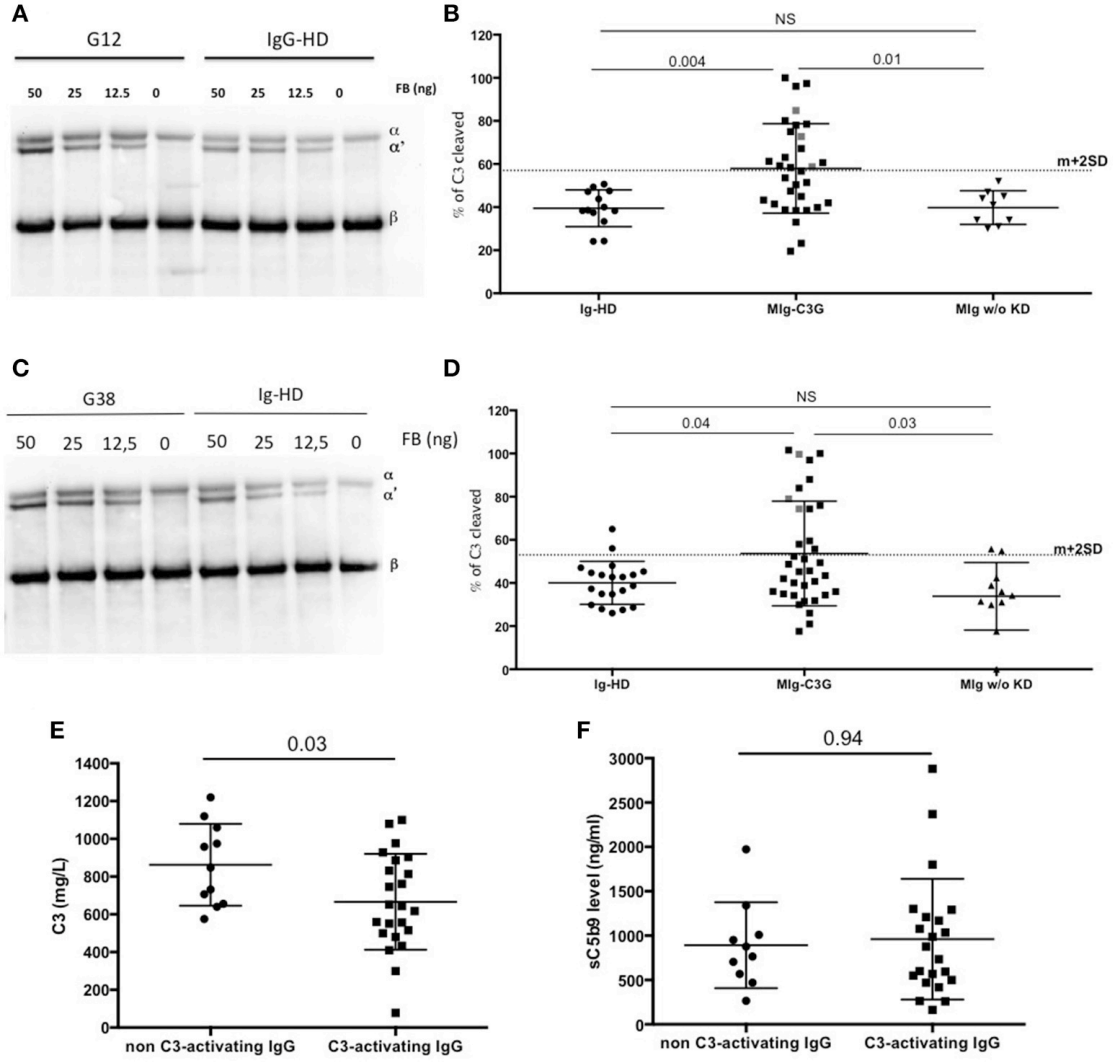
AP convertase activation in presence of patients' total purified IgG. **(A)** MIg-C3G patients' IgG were tested for their capacity to enhance fluid phase AP C3 convertase formation. Cleavage of C3 to C3b by fluid phase C3 convertase was measured by the generation of the α' chain. Result of patient G12 is provided compared to Healthy donor (HD) **(B)** C3 convertase activity was significantly increased in presence of MIg-C3G patients' IgG compared to Ig from HD or Ig from patients with MIg without kidney disease (MIg w/o KD). In presence of Ig from 12/34 patients, % of C3 cleavage was significantly increased [above the cut-off (mean+2SD)]. **(C)** MIg-C3G patients' IgG coated on well plates were tested for their capacity to enhance AP C3 convertase formation. Cleavage of C3–C3b by fluid phase C3 convertase was measured by the generation of the α' chain. Result of patient G38 is provided compared to HD **(D)** C3 convertase activity was significantly increased in presence of MIg-C3G patients' IgG compared to Ig from healthy donors or Ig from patients with MIg without kidney disease (MIg w/o KD). IgG from patients able to enhance C3 convertase in fluid phase or on well plate were named “C3-activating” Ig **(E)** C3 level of patients with “C3-activating” IgG was significantly increased compared to patients without “C3-activating” IgG. **(F)** sC5b9 level of patients with “C3-activating” IgG was similar to patients without “C3-activating” IgG.

To demonstrate that IgG of MIg-C3G patients directly enhance the C3 cleavage into C3b without the influence of auto-antibodies, purified C3, FB, FD were incubated with total IgG from controls (Healthy-donors (HD) and patients with MIg without kidney disease) and MIg-C3G patients and tested in solution or on IgG-coated plate in presence of EGTA-Mg2+. The % of C3 cleavage into C3b was determined by Western blot, by measuring the ratio between α′chain and βchain of C3b, determined by densitometry. Mean % of C3 cleavage was 38% in the presence of IgG from HDs in solution (mean + 2DS of the ratio = 56%) and 39% on HD-IgG-coated plate (mean + 2DS of the ratio = 59%). Cleavage of C3 was increased (higher than mean+2SD) in presence of 12/34 MIg-C3G patients' IgG in solution (Figures 4A,B) and on 13/34 IgG-coated plates (Figures 4C,D). Total IgG purified from 3 patients increased C3b formation both in solution and on coated IgG. Patients' IgG that activated the C3 convertase in solution or on coated phase were named “C3-activating IgG.” Altogether 22/34 tested patients' IgG displayed capacity to cleave C3. In both experimental conditions, C3b formation was significantly higher compared to that obtained in presence of total IgG from patients with MIg but without kidney disease (Figures 4B,D). C3 cleavage was similar in MIg-C3G patients with or without anti-complement protein antibodies (Supplemental Figures 3C,D).

C3 levels were significantly lower in patients positive for C3-activating IgG than in those negative (*P* = 0.03) (Figure 4E), whereas there was no difference for sC5b9 in plasma (*p* = 0.94) (Figure 4F). Plasma sC5b9 levels were upper than twice the normal value in 12/22 (57%) patients with C3-activating IgG and in 3/14 (21%) patients without this capacity (*p* = 0.04).

### Monoclonal Ig are able to enhance fluid phase C3 convertase overactivation

**Figure 5:**
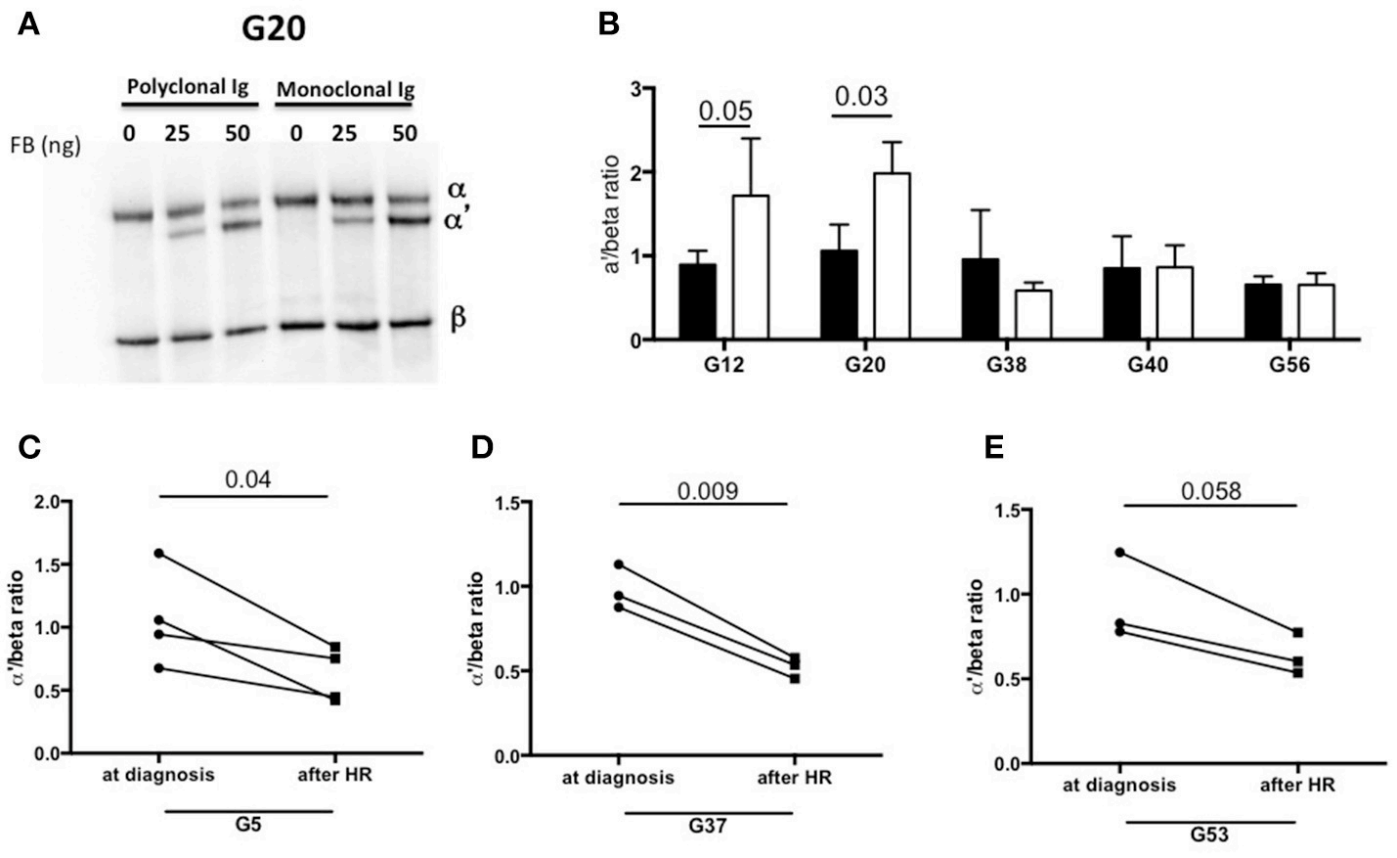
Monoclonal Ig promotes fluid phase C3 convertase activation. **(A)** Polyclonal and monoclonal Ig fractions of 5 MIg-C3G patients were tested for their capacity to enhance fluid phase AP C3 convertase. Cleavage of C3 to C3b by C3 convertase was indicated by generation of the α' chain. **(B)** In 2 out 5 patients (G12, G20), C3 cleavage was increased in presence of the monoclonal Ig fraction (white bars) compared to polyclonal fraction (black bars) (*n* = 3 experiments). In the samples with C3-activating IgG on coated plate, negative for the tests in solution (G38 and G40) and “non C3-activating IgG (G56), C3 convertase activity was similar in presence of the monoclonal and polyclonal Ig fractions. **(C–E)** C3 cleavage by a fluid phase C3 convertase was decreased in 3 patients with “fluid phase activator ” Ig (G5, G37, G53) in presence of Ig purified from plasma collected after hematological response (HR) compared to Ig purified from plasma collected at diagnosis of C3G (*n* = 3 experiments).

To identify the components involved in the AP activation, the MIg was separated from polyclonal Ig by chromatography in 2 patients with C3-activating IgG (G12, G20) and 3 patients without C3 activating IgG in fluid phase (G38, G40, G24). In samples from patients G12 and G20, C3b formation was increased in presence of the MIg compared to the polyclonal Ig (Figures 5A,B).

We also investigated whether the capacity of total IgG to activate the AP disappeared after complete hematological response (as assessed by negative serum immunofixation) following chemotherapy. Blood samples from 3 patients (G5, G37, G53) in whom total IgG were responsible for C3 cleavage in solution were available. In all three cases, C3 cleavage was significantly reduced in presence of total IgG purified from blood after treatment compared to that obtained with IgG from the same patients at diagnosis (Figures 5C–E).

## Discussion

We described for the first time the mechanisms of complement alternative pathway dysregulation in a peculiar group of patients with C3G associated with monoclonal immunoglobulin (MIg-C3G). We found anti complement antibodies in more than 50% of patients but with different target compare to C3G patients without monoclonal gammopathy suggesting that the two diseases are distinct. Moreover, our results highlight a contribution of both monoclonal and polyclonal Ig in the inappropriate activation of complement AP in MIg-C3G patients, paving the way to new therapeutic strategies.

In the French cohort of adult C3G without detectable MIg, impaired complement control is driven by C3NeF and by genetic variation in complement genes in 45 and 27%, respectively. Genetic abnormalities were identified in only 7% of tested MIg-C3G patients, suggesting that genetic factors do not play a major role in MIg-C3G. This result is in agreement with those of a recent study in which none of 21 tested patients had any genetic abnormalities (18). Exhaustive screening identified auto-antibodies targeting complement proteins in about 50% of MIg-C3G patients. However, the targets of anti-complement auto-antibodies were different between C3G patients with and without MIg. Indeed, C3NeF was found in only 7% of MIg-C3G patients. This is in agreement with previous small cohort studies, which identified C3NeF in 0/6 and 2/9 MIg-C3G patients (14, 15). The presence of C5NeF stabilizing the C5 convertase has been recently described in 56% of patients with C3GN (6). Interestingly, despite elevated sC5b-9 level in 80% of MIg-C3G cases, C5NeF was negative in all tested patients. The frequency of anti-FH auto-antibodies was low and similar to C3G patients without MIg (11, 12). In contrast, we found that 27% of MIg-C3G patients had anti-CR1 auto-antibodies, undetectable in C3G patients without MIg. Interestingly, CR1 which is expressed by podocytes, emerges as a novel disease-relevant target in C3G (19) and auto-antibodies targeting CR1 have been found in patients with multiple myeloma (20). We further explored functional consequences of these antibodies. Cofactor activity of both CR1 and FH was decreased in 4/7 and 3/9 patients positive for anti-FH or anti-CR1 antibodies, respectively, whereas it was normal in two patients with anti-FI antibodies suggesting that these antibodies have limited functional consequences on AP regulation. C3 and sC5B9 levels were similar in patients with or without anti-complement protein antibodies, confirming the weak contribution of these antibodies in AP dysregulation in MIg-C3G.

The initial assumption was that autoantibodies targeting complement proteins were monoclonal. Indeed, in 1999, Jokiranta et al. demonstrated that a dimeric monoclonal lambda LC, identified in a patient with glomerulonephritis and predominant C3 deposits, was able to bind FH as an auto-antibody, resulting in uncontrolled AP activation *in vitro* (21). In the current study, we showed a concordance in the heavy and light chain isotypes of MIg and anti-complement protein auto-antibodies in only 3/13 patients. Therefore, our results suggest that in most cases anti-complement protein reactivity is not borne by the MIg. This result is in agreement with other kidney diseases mediated by auto-antibodies, where the implication of monoclonal autoantibodies remains exceptional (22, 23).

Further, we tested a new hypothesis according to which the MIg could serve directly as a complement-activating surface. We designed an experiment to study C3 cleavage without interference with the regulatory proteins and thus without the contribution of anti-complement protein antibodies. In 22/34 (65%) of cases, patients' IgG enhanced C3 cleavage, and therefore they could be considered as C3-activating IgG. Interestingly, C3 level was significantly lower in patients with C3-activating IgG than in those without. Moreover, the percentage of patients with sC5b9 levels higher than twice the normal value was significantly increased in patients with C3-activating IgG compared to those patients without C3-activating IgG. Interestingly, the capacity of patients' IgG to enhance C3 cleavage was not increase in patients with MIg but without kidney disease and the link between an ongoing complement activation in MIg-C3G patients and the MIg remains speculative. The direct role of the MIg in AP activation was strongly suggested in 5 patients. Indeed, in 3 of them, we demonstrated the disappearance of the capacity of total IgG to activate the C3 convertase once the MIg had become undetectable after chemotherapy. In 2 patients, we showed the increased capacity of the MIg to enhance fluid phase C3 convertase activity compared to the polyclonal IgG from the same patients. It is well established that MIg have peculiar physicochemical properties due to different profiles of glycosylation or mutations/deletions of the variable or constant domain (24). These peculiarities are likely to account for the variable capacity of these MIg to enhance C3 convertase *in vitro*. It is tempting to speculate that the nascent C3b, generated by slow fluid phase activation of C3, binds to MIg and forms a starting point for the subsequent assembly of C3 convertase.

In a recent clinical study, we demonstrated that achievement of rapid and deep hematological response with clone-targeted chemotherapy significantly improved renal survival in MIg-C3G patients and that C3 levels in patients with hematological response were significantly higher compared with pretreatment C3 levels (16). The present provides more support for a link between the monoclonal Ig and renal disease. Therefore targeting the responsible clone should be a therapeutic goal to preserve or improve renal function in these patients.

Our study has some limitations. It is a retrospective study with a relatively low number of patients. Most patients had low amounts of MIg making the MIg purification process difficult or even impossible. These limitations did not allow us to investigate the direct contribution of MIg in AP dysregulation in all patients, and further studies are needed to depict the full pathophysiological spectrum of MIg in C3GP.

In conclusion, our study highlight different complement AP activation mechanisms in C3G associated with MIg compared to C3G without MIg. We demonstrated that IgG isolated from MIg-C3G patients directly activate the AP in 65% of cases and our findings provide further evidence that monoclonal gammopathy is a cause of the disease, particularly in patients with very high levels of sC5b9 at diagnosis. Our results highlight the need to consider chemotherapy targeting the B cell clone in the treatment strategy of MIg-C3G patients.

## Author contributions

The study was conceived and designed by SC and VF-B. SC conducted the experiments and analysis; SC and VF-B were involved in the writing of the manuscript. VF-B and LR reviewed the data analysis; SC, VF-B, FB, and all other authors contributed to the conduct of the study, recruited patients, and were involved in the review of results and final approval of the manuscript.

## Disclosure

VF-B received fees for participation in advisory boards, experts meetings and/or teaching courses from Alexion Pharmaceutical. YD received honoraria from Alexion Pharmaceutical for teaching symposia.

### Conflict of interest statement

The authors declare that the research was conducted in the absence of any commercial or financial relationships that could be construed as a potential conflict of interest. The reviewer GR declared a past co-authorship with two of the authors: VF and PR.
