# Supplementary material for: Both Monoclonal and Polyclonal Immunoglobulin Contingents Mediate Complement Activation in Monoclonal Gammopathy Associated-C3 Glomerulopathy
Source: Front Immunol. 2018 Oct 2;9:2260. doi: 10.3389/fimmu.2018.02260 (PMC6175995; doi:10.3389/fimmu.2018.02260)
Supplement: Supplementary file 1 [file Presentation_1.PPTX]

## Slide 1
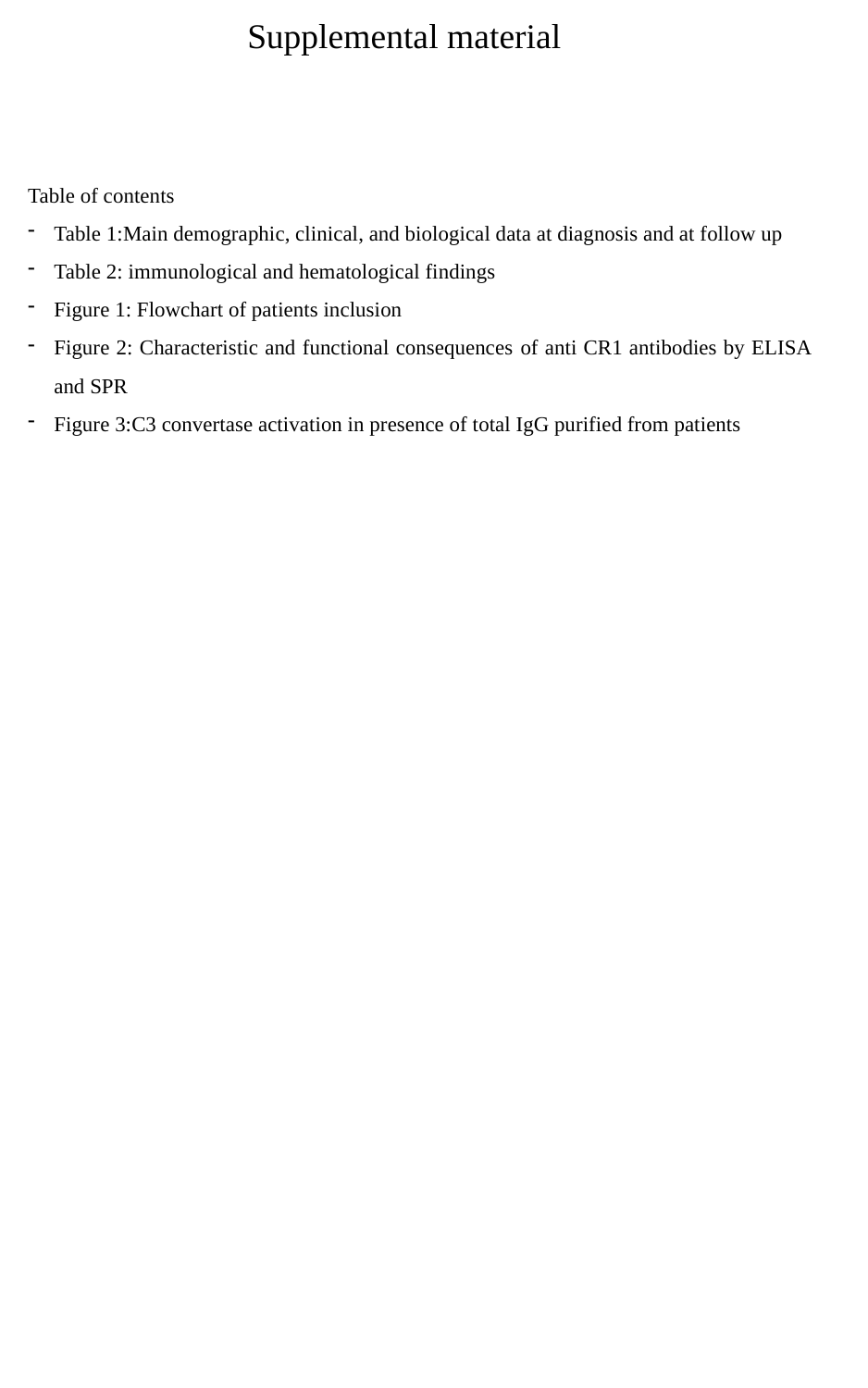

Supplemental material
Table of contents
Table 1:Main demographic, clinical, and biological data at diagnosis and at follow up
Table 2: immunological and hematological findings
Figure 1: Flowchart of patients inclusion
Figure 2: Characteristic and functional consequences of anti CR1 antibodies by ELISA and SPR
Figure 3:C3 convertase activation in presence of total IgG purified from patients

## Slide 2
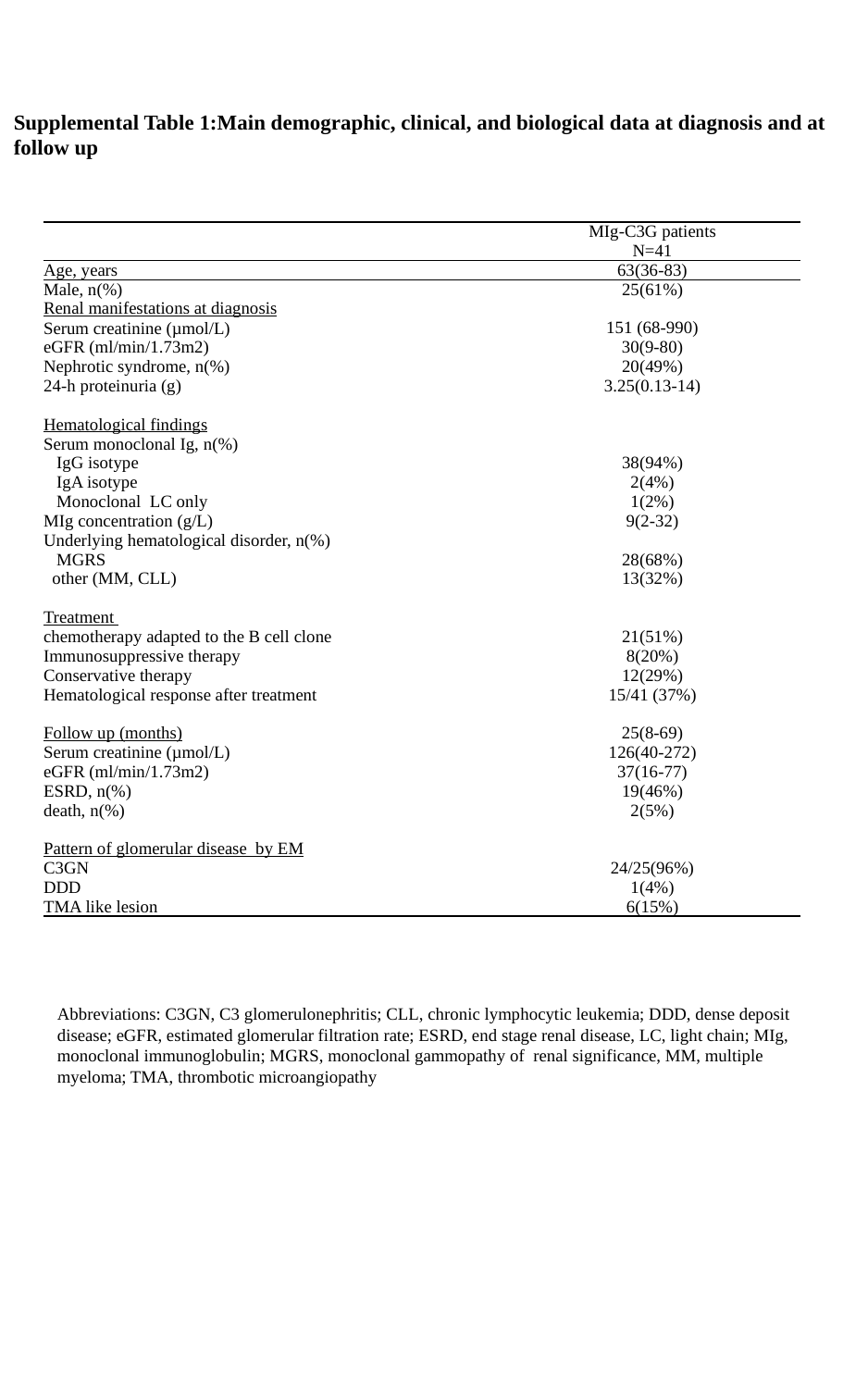

Supplemental Table 1:Main demographic, clinical, and biological data at diagnosis and at follow up
| | MIg-C3G patients |
| --- | --- |
| | N=41 |
| Age, years | 63(36-83) |
| Male, n(%) | 25(61%) |
| Renal manifestations at diagnosis | |
| Serum creatinine (µmol/L) | 151 (68-990) |
| eGFR (ml/min/1.73m2) | 30(9-80) |
| Nephrotic syndrome, n(%) | 20(49%) |
| 24-h proteinuria (g) | 3.25(0.13-14) |
| | |
| Hematological findings | |
| Serum monoclonal Ig, n(%) | |
| IgG isotype | 38(94%) |
| IgA isotype | 2(4%) |
| Monoclonal LC only | 1(2%) |
| MIg concentration (g/L) | 9(2-32) |
| Underlying hematological disorder, n(%) | |
| MGRS | 28(68%) |
| other (MM, CLL) | 13(32%) |
| | |
| Treatment | |
| chemotherapy adapted to the B cell clone | 21(51%) |
| Immunosuppressive therapy | 8(20%) |
| Conservative therapy | 12(29%) |
| Hematological response after treatment | 15/41 (37%) |
| | |
| Follow up (months) | 25(8-69) |
| Serum creatinine (µmol/L) | 126(40-272) |
| eGFR (ml/min/1.73m2) | 37(16-77) |
| ESRD, n(%) | 19(46%) |
| death, n(%) | 2(5%) |
| | |
| Pattern of glomerular disease by EM | |
| C3GN | 24/25(96%) |
| DDD | 1(4%) |
| TMA like lesion | 6(15%) |
Abbreviations: C3GN, C3 glomerulonephritis; CLL, chronic lymphocytic leukemia; DDD, dense deposit disease; eGFR, estimated glomerular filtration rate; ESRD, end stage renal disease, LC, light chain; MIg, monoclonal immunoglobulin; MGRS, monoclonal gammopathy of renal significance, MM, multiple myeloma; TMA, thrombotic microangiopathy

## Slide 3
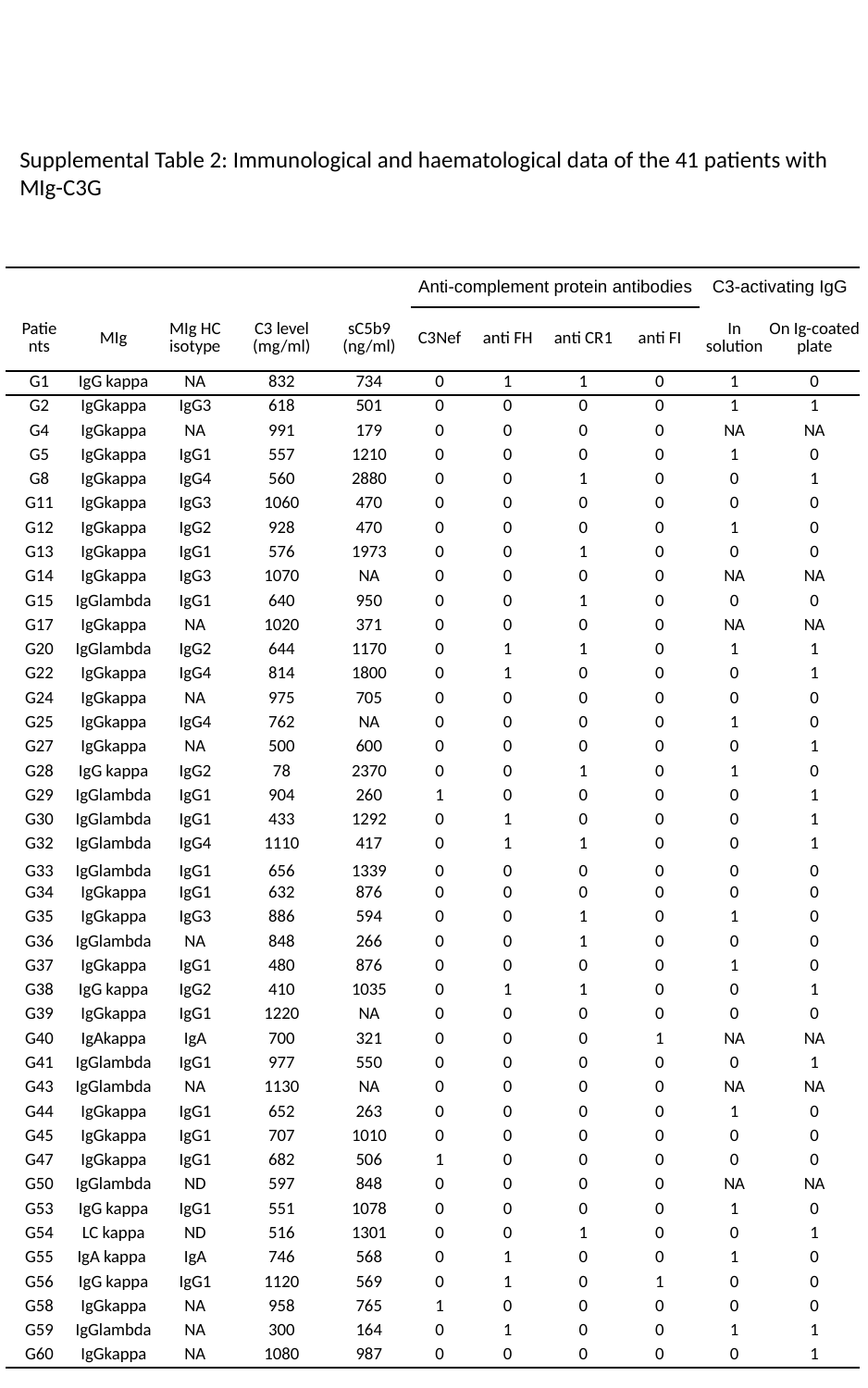

Supplemental Table 2: Immunological and haematological data of the 41 patients with MIg-C3G
| | | | | | Anti-complement protein antibodies | | | | C3-activating IgG | |
| --- | --- | --- | --- | --- | --- | --- | --- | --- | --- | --- |
| Patients | MIg | MIg HC isotype | C3 level (mg/ml) | sC5b9 (ng/ml) | C3Nef | anti FH | anti CR1 | anti FI | In solution | On Ig-coated plate |
| G1 | IgG kappa | NA | 832 | 734 | 0 | 1 | 1 | 0 | 1 | 0 |
| G2 | IgGkappa | IgG3 | 618 | 501 | 0 | 0 | 0 | 0 | 1 | 1 |
| G4 | IgGkappa | NA | 991 | 179 | 0 | 0 | 0 | 0 | NA | NA |
| G5 | IgGkappa | IgG1 | 557 | 1210 | 0 | 0 | 0 | 0 | 1 | 0 |
| G8 | IgGkappa | IgG4 | 560 | 2880 | 0 | 0 | 1 | 0 | 0 | 1 |
| G11 | IgGkappa | IgG3 | 1060 | 470 | 0 | 0 | 0 | 0 | 0 | 0 |
| G12 | IgGkappa | IgG2 | 928 | 470 | 0 | 0 | 0 | 0 | 1 | 0 |
| G13 | IgGkappa | IgG1 | 576 | 1973 | 0 | 0 | 1 | 0 | 0 | 0 |
| G14 | IgGkappa | IgG3 | 1070 | NA | 0 | 0 | 0 | 0 | NA | NA |
| G15 | IgGlambda | IgG1 | 640 | 950 | 0 | 0 | 1 | 0 | 0 | 0 |
| G17 | IgGkappa | NA | 1020 | 371 | 0 | 0 | 0 | 0 | NA | NA |
| G20 | IgGlambda | IgG2 | 644 | 1170 | 0 | 1 | 1 | 0 | 1 | 1 |
| G22 | IgGkappa | IgG4 | 814 | 1800 | 0 | 1 | 0 | 0 | 0 | 1 |
| G24 | IgGkappa | NA | 975 | 705 | 0 | 0 | 0 | 0 | 0 | 0 |
| G25 | IgGkappa | IgG4 | 762 | NA | 0 | 0 | 0 | 0 | 1 | 0 |
| G27 | IgGkappa | NA | 500 | 600 | 0 | 0 | 0 | 0 | 0 | 1 |
| G28 | IgG kappa | IgG2 | 78 | 2370 | 0 | 0 | 1 | 0 | 1 | 0 |
| G29 | IgGlambda | IgG1 | 904 | 260 | 1 | 0 | 0 | 0 | 0 | 1 |
| G30 | IgGlambda | IgG1 | 433 | 1292 | 0 | 1 | 0 | 0 | 0 | 1 |
| G32 | IgGlambda | IgG4 | 1110 | 417 | 0 | 1 | 1 | 0 | 0 | 1 |
| G33 | IgGlambda | IgG1 | 656 | 1339 | 0 | 0 | 0 | 0 | 0 | 0 |
| G34 | IgGkappa | IgG1 | 632 | 876 | 0 | 0 | 0 | 0 | 0 | 0 |
| G35 | IgGkappa | IgG3 | 886 | 594 | 0 | 0 | 1 | 0 | 1 | 0 |
| G36 | IgGlambda | NA | 848 | 266 | 0 | 0 | 1 | 0 | 0 | 0 |
| G37 | IgGkappa | IgG1 | 480 | 876 | 0 | 0 | 0 | 0 | 1 | 0 |
| G38 | IgG kappa | IgG2 | 410 | 1035 | 0 | 1 | 1 | 0 | 0 | 1 |
| G39 | IgGkappa | IgG1 | 1220 | NA | 0 | 0 | 0 | 0 | 0 | 0 |
| G40 | IgAkappa | IgA | 700 | 321 | 0 | 0 | 0 | 1 | NA | NA |
| G41 | IgGlambda | IgG1 | 977 | 550 | 0 | 0 | 0 | 0 | 0 | 1 |
| G43 | IgGlambda | NA | 1130 | NA | 0 | 0 | 0 | 0 | NA | NA |
| G44 | IgGkappa | IgG1 | 652 | 263 | 0 | 0 | 0 | 0 | 1 | 0 |
| G45 | IgGkappa | IgG1 | 707 | 1010 | 0 | 0 | 0 | 0 | 0 | 0 |
| G47 | IgGkappa | IgG1 | 682 | 506 | 1 | 0 | 0 | 0 | 0 | 0 |
| G50 | IgGlambda | ND | 597 | 848 | 0 | 0 | 0 | 0 | NA | NA |
| G53 | IgG kappa | IgG1 | 551 | 1078 | 0 | 0 | 0 | 0 | 1 | 0 |
| G54 | LC kappa | ND | 516 | 1301 | 0 | 0 | 1 | 0 | 0 | 1 |
| G55 | IgA kappa | IgA | 746 | 568 | 0 | 1 | 0 | 0 | 1 | 0 |
| G56 | IgG kappa | IgG1 | 1120 | 569 | 0 | 1 | 0 | 1 | 0 | 0 |
| G58 | IgGkappa | NA | 958 | 765 | 1 | 0 | 0 | 0 | 0 | 0 |
| G59 | IgGlambda | NA | 300 | 164 | 0 | 1 | 0 | 0 | 1 | 1 |
| G60 | IgGkappa | NA | 1080 | 987 | 0 | 0 | 0 | 0 | 0 | 1 |

## Slide 4
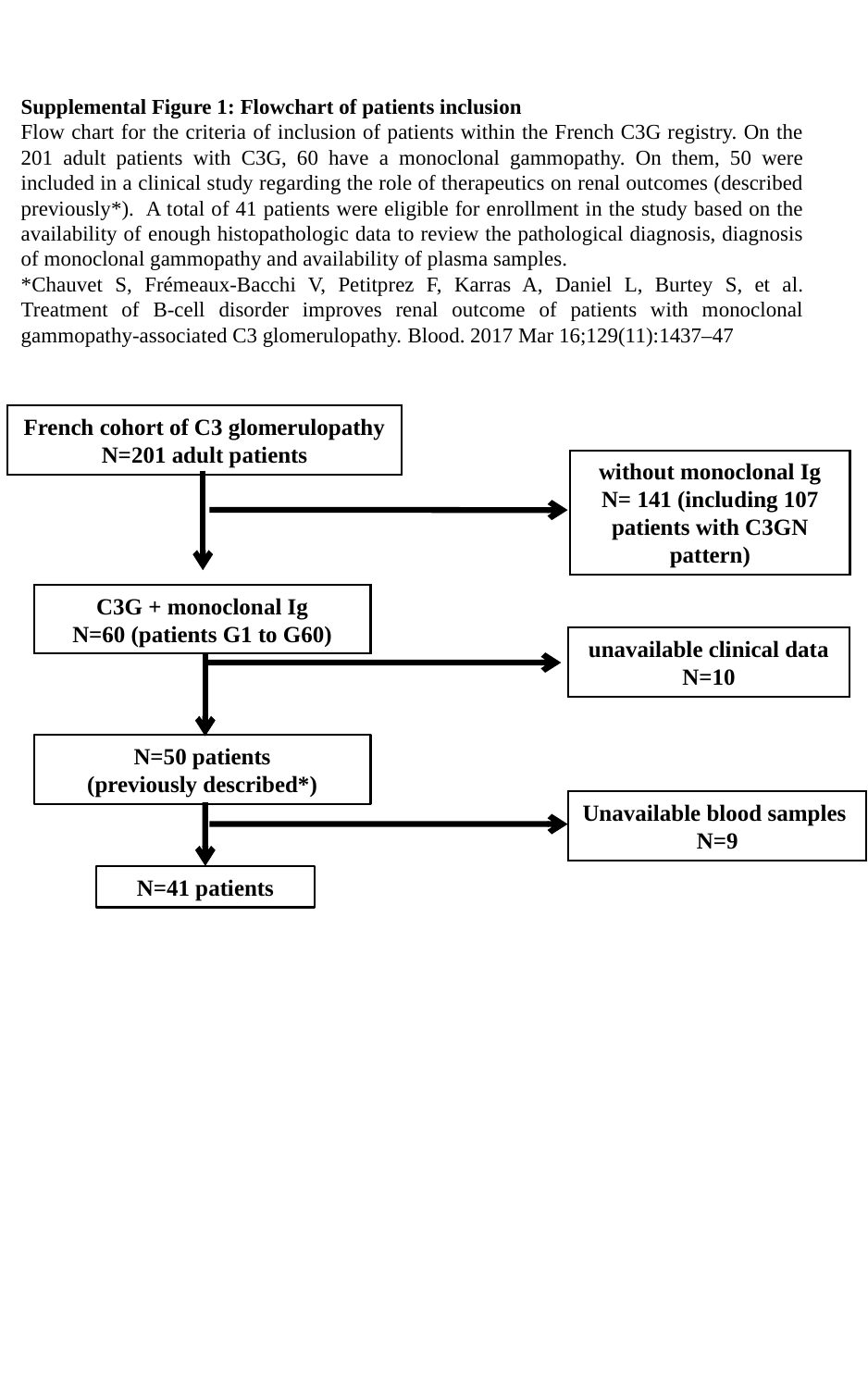

Supplemental Figure 1: Flowchart of patients inclusion
Flow chart for the criteria of inclusion of patients within the French C3G registry. On the 201 adult patients with C3G, 60 have a monoclonal gammopathy. On them, 50 were included in a clinical study regarding the role of therapeutics on renal outcomes (described previously*). A total of 41 patients were eligible for enrollment in the study based on the availability of enough histopathologic data to review the pathological diagnosis, diagnosis of monoclonal gammopathy and availability of plasma samples.
*Chauvet S, Frémeaux-Bacchi V, Petitprez F, Karras A, Daniel L, Burtey S, et al. Treatment of B-cell disorder improves renal outcome of patients with monoclonal gammopathy-associated C3 glomerulopathy. Blood. 2017 Mar 16;129(11):1437–47
French cohort of C3 glomerulopathy
N=201 adult patients
without monoclonal Ig
N= 141 (including 107 patients with C3GN pattern)
C3G + monoclonal Ig
N=60 (patients G1 to G60)
unavailable clinical data
N=10
N=50 patients
(previously described*)
Unavailable blood samples
N=9
N=41 patients

## Slide 5
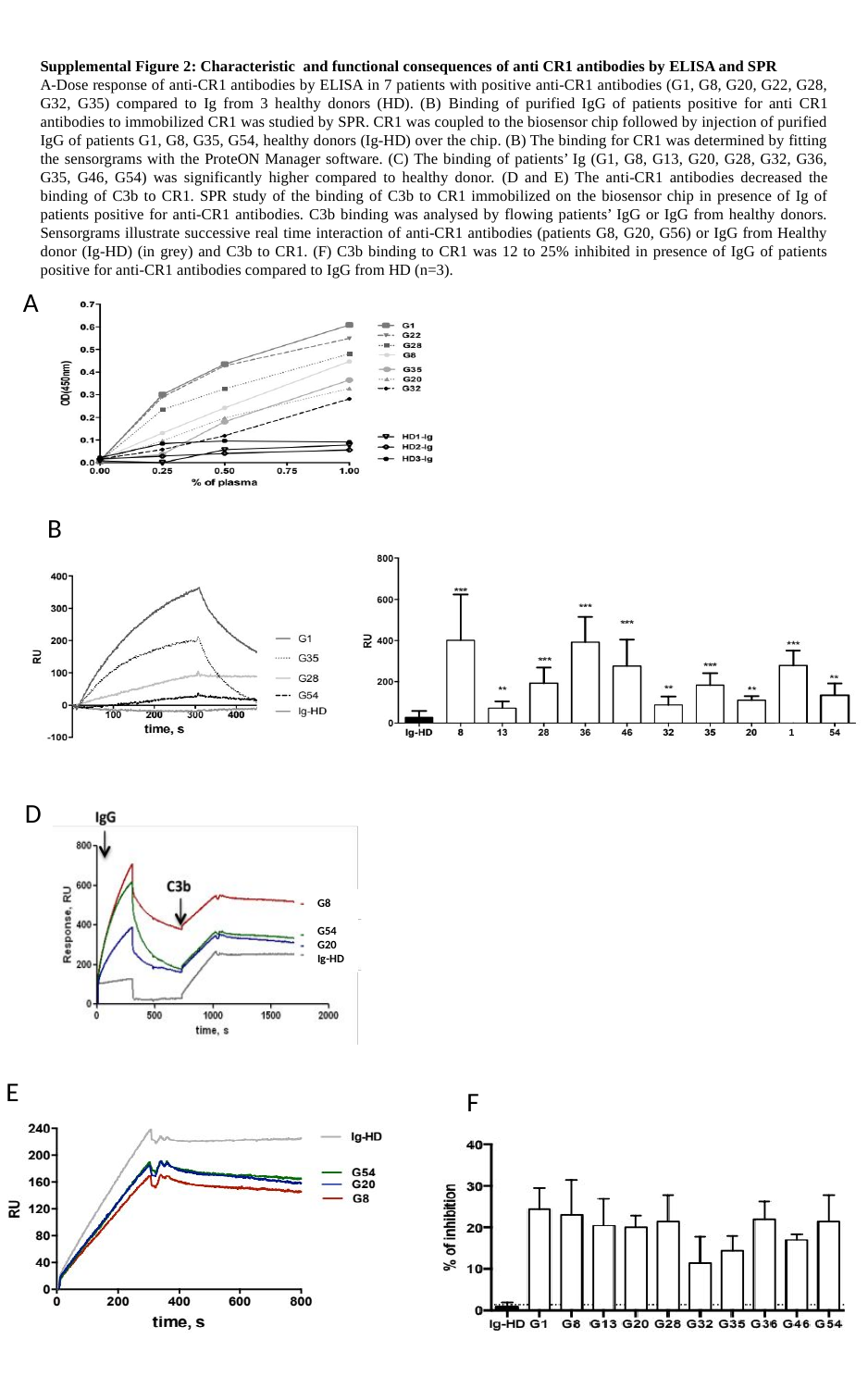

Supplemental Figure 2: Characteristic and functional consequences of anti CR1 antibodies by ELISA and SPR
A-Dose response of anti-CR1 antibodies by ELISA in 7 patients with positive anti-CR1 antibodies (G1, G8, G20, G22, G28, G32, G35) compared to Ig from 3 healthy donors (HD). (B) Binding of purified IgG of patients positive for anti CR1 antibodies to immobilized CR1 was studied by SPR. CR1 was coupled to the biosensor chip followed by injection of purified IgG of patients G1, G8, G35, G54, healthy donors (Ig-HD) over the chip. (B) The binding for CR1 was determined by fitting the sensorgrams with the ProteON Manager software. (C) The binding of patients’ Ig (G1, G8, G13, G20, G28, G32, G36, G35, G46, G54) was significantly higher compared to healthy donor. (D and E) The anti-CR1 antibodies decreased the binding of C3b to CR1. SPR study of the binding of C3b to CR1 immobilized on the biosensor chip in presence of Ig of patients positive for anti-CR1 antibodies. C3b binding was analysed by flowing patients’ IgG or IgG from healthy donors. Sensorgrams illustrate successive real time interaction of anti-CR1 antibodies (patients G8, G20, G56) or IgG from Healthy donor (Ig-HD) (in grey) and C3b to CR1. (F) C3b binding to CR1 was 12 to 25% inhibited in presence of IgG of patients positive for anti-CR1 antibodies compared to IgG from HD (n=3).
A
B
1
D
G8
G54
G20
Ig-HD
E
F

## Slide 6
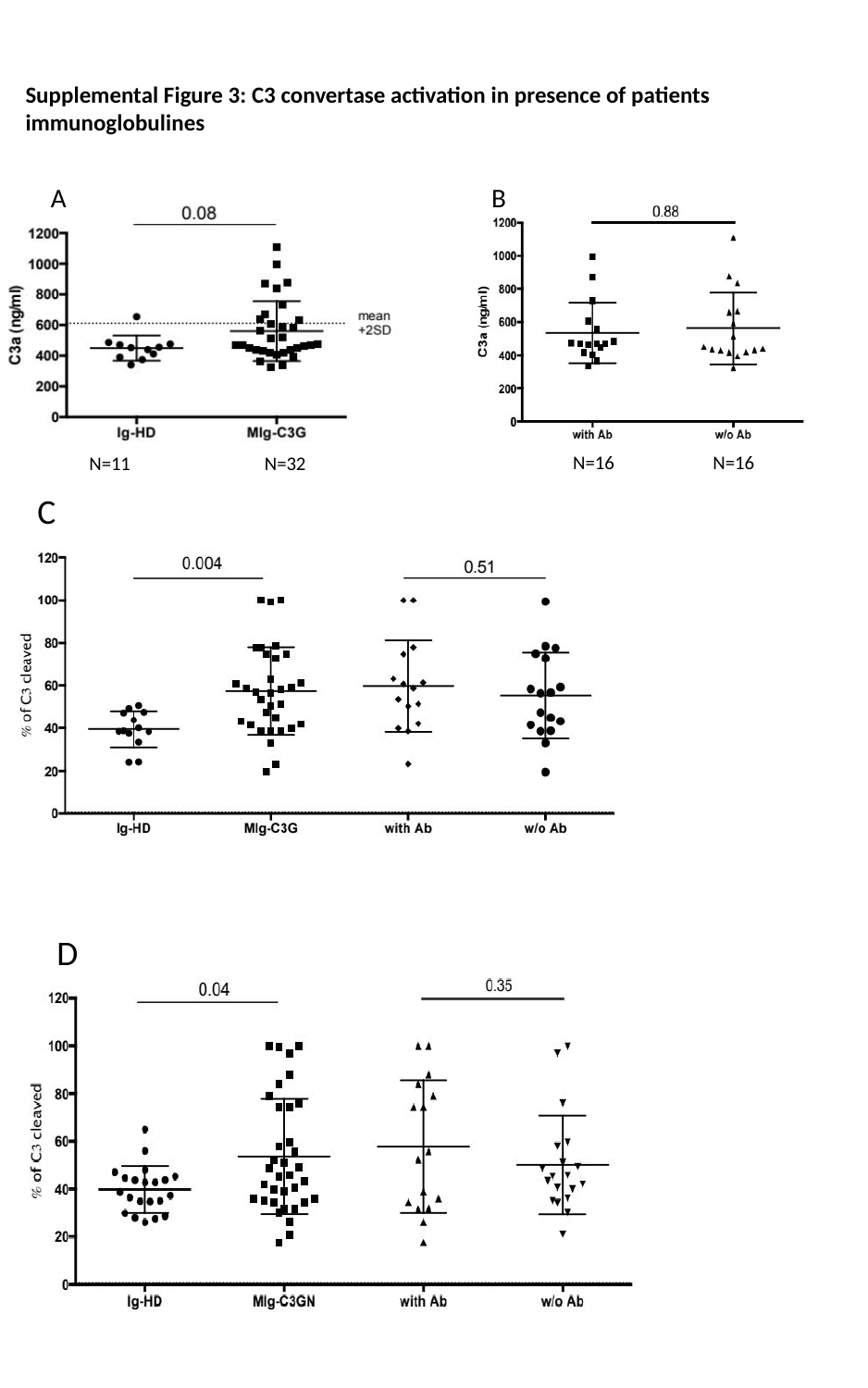

Supplemental Figure 3: C3 convertase activation in presence of patients immunoglobulines
A
B
N=16
N=16
N=32
N=11
C
D
